# A functional study on gentamicin-related cochleotoxicity in its conventional dose in newborns

**DOI:** 10.1590/S1808-86942010000100015

**Published:** 2015-10-17

**Authors:** Carla Luiza Baggio, Aron Ferreira da Silveira, Miguel Angelo Hyppolito, Flávia Fiacadori Salata, Maria Rossato

**Affiliations:** 1 MSc, Human Communication Disorders, UFSM, Speech and Hearing Therapist; 2 PhD, Veterinary Medicine. Professor, Graduate Program on Human Communication Disorders, Professor, Morphology Department, UFSM; 3 PhD, Medical Sciences, USP, MD, ENT, Professor of Ophthalmology, Department of Ophthalmology, Otorhinolaryngology, and HNS, Division of Otorhinolaryngology, Medical School at Ribeirão Preto, University of São Paulo FMRPUSP; 4 Biologist, Assistant Biologist, Hearing Neurobiology Lab, Department of Ophthalmology, Otorhinolaryngology, and HNS, Division of Otorhinolaryngology, Medical School at Ribeirão Preto, University of São Paulo FMRPUSP; 5 Lab Technician, Hearing Neurobiology Lab, Department of Ophthalmology, Otorhinolaryngology, and HNS, Division of Otorhinolaryngology, Medical School at Ribei-rão Preto, University of São Paulo FMRPUSP

**Keywords:** hearing, gentamicin, infant, toxicity

## Abstract

The early identification of hearing impairment allows for an intervention still in the “critical” and ideal period of hearing and language stimulation. Pediatric ototoxicity is a very controversial topic. There have been variable percentages of ototoxicity cases in children with different aminoglycosides antibiotics. The main pediatric groups whom receive aminoglycosides are newborns with severe infections on the neonatal ICU.

**Aim:**

to check the functional aspect of the cochlear external hair cells and treatment regimens used to treat infections during the neonatal period.

**Study design:**

Experimental.

**Materials and Methods:**

we studied 26 albino guinea pigs, through distortion product otoacoustic emissions, before and after the use of gentamicin.

**Results:**

in all the assessments, the external hair cells functional status, studied by means of the distortion product otoacoustic emissions, proved preserved.

**Conclusion:**

In the present study, we did not notice changes in outer hair cell function in the albino guinea pigs treated with gentamicin in the doses of 4 mg/Kg/ day and 2.5 mg/Kg/day every 12 hours for 10 and 14 days.

## INTRODUCTION

Hearing plays a very important role in human communication. Hearing losses may serve as background to psychosocial disorders, as the inability to understand spoken language may sever one's contact with others and the world^1^.

Early identification of hearing loss cases enables intervention still in the 'critical period' in time to stimulate language and hearing. The central auditory system's maturation process occurs during the first years of life. Hearing experiences in this period of greater brain plasticity and establishment of new neuronal connections are indispensable to ensure the development of both hearing and language^2^.

From the standpoint of physiology, the auditory reception structure is formed by support cells and hair cells located in the organ of Corti. The outer hair cells cannot receive sound stimuli, but they can contract quickly or slowly due to their biomechanical properties^3^.

Outer hair cells make up the cochlear amplifier and play an important role in amplifying sound signals and determining the function of inner hair cells, the cochlear reception and encoding units that play an important role in cochlear frequency selectivity^4^.

Vibratory-mechanical signals reaching the cochlea stimulate the outer hair cells. Such stimulation is then amplified towards the inner hair cells. A measurable echo (otoacoustic emission) is then created^5^.

Otoacoustic emissions (OAEs) are low-intensity sound signals amplified by the contraction of outer hair cells in the cochlea, possibly captured in the external acoustic meatus. They were discovered in 1978 by David T. Kemp, Professor of Hearing Biophysics at the London University College. OAEs may be categorized as: spontaneous - when captured at the external acoustic meatus in the absence of acoustic stimulation; evoked - when there is release of energy captured in the external acoustic meatus in response to acoustic stimulation. Evoked OAEs can be classified as transient - evoked by short broad specter acoustic stimulation covering a range of frequencies, clicks or tone burst; distortion product - evoked by two pure simultaneous tones (F1 and F2) which by intermodulation produce a distortion product as a response (2F1-F2); frequency-stimulation, evoked by a continuous signal of low intensity in the frequency of the presented stimulation: these are clinically less used^4^.

Hearing loss may congenital or acquired and manifest itself in many different degrees and types. Among the causes for acquired hearing loss is the use of ototoxic drugs, medication that introduces adverse effects on the structures of the inner ear thus affecting the auditory and/ or vestibular systems.

Pediatric ototoxicity is a controversial topic. Varied prevalence rates have been reported for ototoxicity in children taking various aminoglycosides such as streptomycin, dihydrostreptomycin, kanamycin, amikacin, and gentamicin, the latter being an important drug in the treatment of various infections caused by gram-negative bacilli^6^.

Gentamicin is the first aminoglycoside of choice because of its low cost and as it is active in most resistant aerobic gram-negative bacilli7. The main pediatric groups receiving aminoglycosides are neonates with severe infection staying at the neonatal ICU^6^.

Aminoglycosides damage the outer hair cells by initially compromising the cochlear basal turn cells. The incidence of ototoxicity by gentamicin ranges from 6% to 16% ^8^.

Animal studies have played an essential role in the development of Science. Experimental research with animal models is important in developing new drugs, enhancing the knowledge on disease pathophysiological mechanisms, understanding treatment modes with new drugs, studying biologic markers, and assessing techniques to be applied in humans in the future^9^.

Expanding the knowledge on this area will enable health care workers to adopt procedures that look at maintaining the auditory integrity of their patients, consequently allowing them to enjoy a better life.

This study analyzed findings from the literature and looked into the dosages of gentamicin applied in the neonatal ICU setting to verify the relationship between outer hair cell function and neonatal infection therapies.

## MATERIALS AND METHOD

Twenty-six female, albino Guinea pigs weighing between 400 and 500 grams with normal Preyer reflex were used in this study. All animals were used in accordance with the guidelines of our institution, which by their turn were based on the guide for the care of lab animals by the Institute of Laboratory Animal Resources, Commission on Life Sciences, National Research council, National Academy Press, Washington, D. C., 1996. This study was approved by the Animal Experimentation Ethics Committee under permit 073/2007.

The drug used in this study was aminoglycoside gentamicin (Geramicin, 60 mg/ml, Schering) by intramuscular administration. All animals were weighed every other Day to control medication dosage. Guinea pigs were assessed for distortion product otoacoustic emissions in a soundproof booth under anesthesia by ketamine (Ketamin 50mg/ml, Cristália,) 40 mg/kg and xylazine (Dopaser 20 mg/ml, Calier do Brasil,) 10 mg/kg, pre and post gentamicin administration. The equipment used was the ILO 92 CAE System Otodynamics LTD.

Guinea pigs were divided into five groups:

• Group 1 - control group (placebo) - two subjects (four cochleae) given saline solution 1.0 ml every 12 hours by intramuscular injection for 14 days;
• Group 2: six subjects (12 cochleae) given gentamicin 4 mg/kg/day for 10 days;
• Group 3: six subjects (12 cochleae) given gentamicin 4 mg/kg/day for 14 days;
• Group 4: six subjects (12 cochleae) given gentamicin 2.5 mg/kg/Day every 12 hours for 10 days;
• Group 5: six subjects (12 cochleae) given gentamicin 2.5 mg/kg/Day every 12 hours for 14 days.

This study considered otoacoustic emissions starting at 1500 Hz to assess the functional status of outer hair cells in the cochlear basal turn, looking at the signal/noise ratio of the emissions. Statistical analysis used at first the Shapiro-Wilk test. As the data sets did not follow a normal distribution pattern we opted to use non-parametric tests: variable dependent (Wilcoxon) and independent (Kruskal Wallis). Significance level was set at 5%.

## RESULTS

Considering the used dosage of gentamicin of 4 mg/Kg/Day for 10 days of consecutive treatment (group 2), no changes were observed in the distortion product otoacoustic before and after treatment in qualitative analysis, as otoacoustic emissions were present in both circumstances.

**Figure 1:**
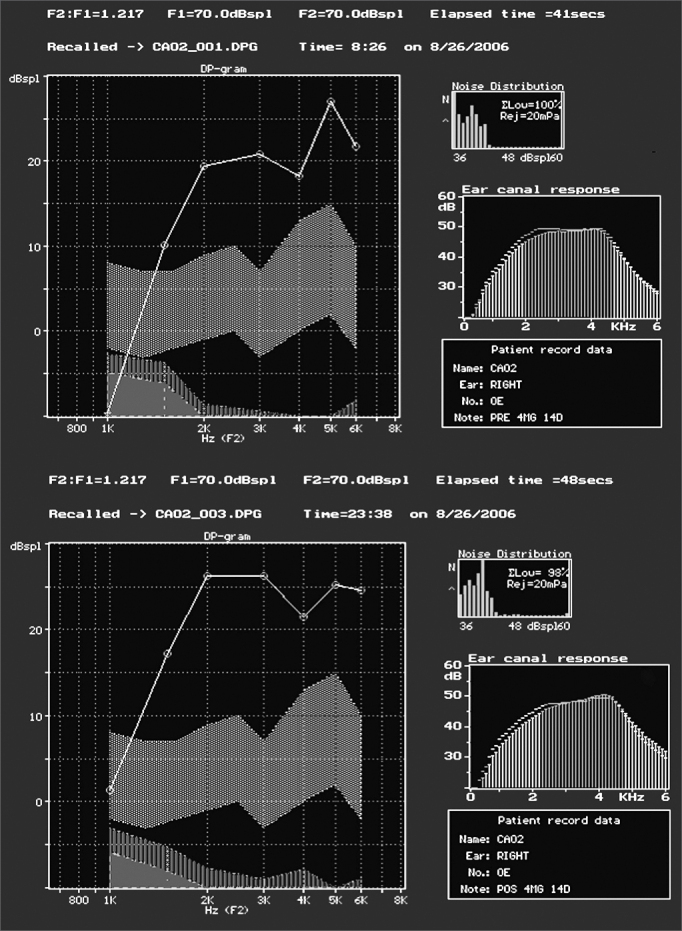
Screenshot of program ILO 92 in which we can see the presence of response to distortion product otoacoustic emissions in the group treated with gentamicin 4 mg/Kg/day for 14 days, before (A) and after (B) treatment (on the day they were slaughtered).

Subjects on Group 3, treated with gentamicin 4 mg/Kg/day for 14 days, did not have changes in their distortion product otoacoustic emissions after treatment. Figure 1 shows an example of present distortion product otoacoustic emissions before and after treatment for Group 3 and also observed in all other studied groups. Groups 4 - treated with gentamicin 5 mg/Kg/day for 10 days - and 5 - gentamicin 2.5 mg/Kg/day every 12 hours by intra-peritoneal administration for 14 days, distortion product OAEs were present before and after treatment.

Distortion product OAEs starting at 1.5 kHz were deemed present, as the size of the external ear meatus of Guinea pigs makes it difficult for OAEs below this frequency to be detected, producing responses coinciding with responses to noise.

In all tests the functional status of outer hair cells, as analyzed by distortion product OAEs, was preserved.

The non-parametric Kruskal-Wallis test was used to compare the signal/noise ratios before and after treatment with gentamicin for statistical purposes.

**Figure 2:**
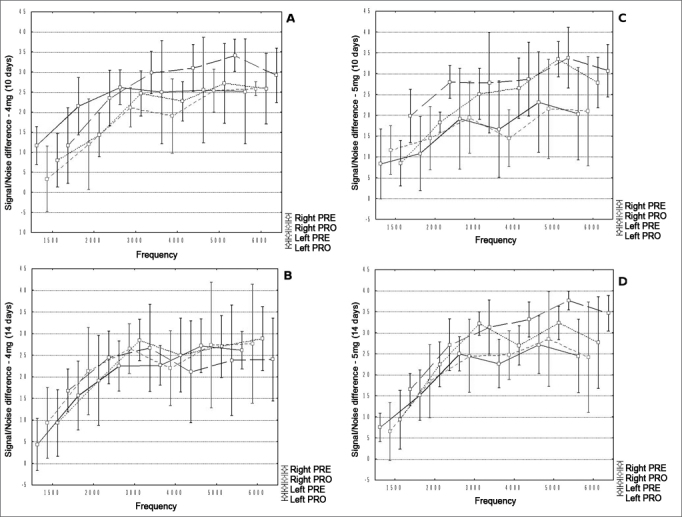
A, B, C and D. Distribution of the signal/noise ratios (Y) in dB NPS and studied frequencies to distortion product otoacoustic emissions (X) in Hz for various studied groups (2, 3, 4, and 5 respectively).

No statistically significant differences were found among groups (p>0.05). The data on the signal/noise ratios observed in Groups 2, 3, 4, and 5 are shown in Figures 2 A, B, C and D respectively.

## DISCUSSION

After conducting an experiment using distortion product otoacoustic emissions of albino Guinea pigs treated with gentamicin in order to verify outer hair cell function, we found that the results obtained here support the findings of other studies carried out on the matter.

There is no doubt that highly sensitive and specific auditory follow-up must be adopted when there is exposure to ototoxic medication. According to the author, in order for the pediatric population to be monitored it is recommended that otoacoustic emissions are used to find hearing disorders before the more significant frequencies for speech recognition are affected, thus preventing the occurrence of difficulties in psychosocial development and academic life^10^.

Ototoxic drugs may potentially alter cochlear bio-mechanics and affect hearing^11^. OAEs are important in assessing outer hair cell function along the organ of Corti, enabling tonotopic identification of cochlear hair cell injuries. It is useful to monitor risk of hearing impairment^12^.

According to Amatuzzi et al.^13^, the prevalence rates of sensorineural hearing loss in neonates treated at intensive care units ranges from 2% to 4%, or thirteen times greater than that of non-ICU neonates. Risk factors for hearing loss such as congenital infection, neonatal anoxia, hyperbilirubinemia and use of ototoxic drugs have been studied by authors such as Kountakis et al. (2002)^14^ and Hoog et al.^15^.

McCracken apud Matz^16^ looked at the ototoxic potential of streptomycin, kanamycin, and gentamicin in seven prospective studies covering over 1,300 neonates. Results indicated that the risk of neonates clinically developing significant hearing impairment after 3 to 7 days of using aminoglycosides is minimal. The abovementioned author concluded that aminoglycosides have been used with high degrees of safety and efficacy in neonates and children for over two decades, as also found in this study.

Agarwal et al.^17^ carried out a study on the neonatal ICU at the Cook County Children's Hospital IL, Chicago in 1999 and compared the pharmacokinetics of gentamicin 4 mg/Kg single daily dose to 2.5 mg/Kg every 12 hours in children under 7 days old. All patients underwent auditory screening using an ALGO Natus device. Results were either Pass or Fail for each ear. Sensitivity was of 98% and specificity of 96%. All the children underwent hearing acuity tests before discharge. In this study the authors concluded that a daily dose of 4 mg/Kg of gentamicin presented a significantly higher, safe and consistent peak of blood concentration in all subjects compared to the scheme 2.5 mg/Kg every 12 hours.

Gentamicin arrives at the inner ear tissues after systemic administration in concentrations that do not exceed its plasmatic concentration; therefore, it is not actively accumulated in the inner ear. Concentrations are lower in the endolymph than in the perilymph. Within approximately 12 hours systemically administered gentamicin reaches the cochlear fluids, taking about 24 hours to see its levels reduce significantly^18^.

The positive polarity of the endocochlear potential appears to favor the entry of cationic substances such as gentamicin in the scala media. The communication between the vascular system and the cochlear fluids occurs from the spiral ligament capillary beds and the stria vascularis on the lateral wall of the cochlea. The scala vestibuli and scala tympani promptly communicate with the spaces of the spiral ligament, and the endolymphatic space can be reached through the indirect passing of cationic drugs from the scala vestibuli and scala tympani and directly via the stria vascularis^19^.

According to Martins^20^, in terms of neonatal septicemia treatment duration, it is recommended that clinical therapy is offered for seven to fourteen days. Under the dosages proposed in this experiment, no significant alterations were found in the signal/noise ratios of product distortion OAEs, nor any significant alteration in their amplitudes, as also seen in the literature.

The literature is still insufficient to fully explain the ototoxic effects drugs such as gentamicin may have on neonates. Pre and perinatal risk factors associated to the use of ototoxic medication, changes in the renal clearance, and association with multiple drugs increase the toxic potential of gentamicin.

Otologic research frequently requires the use of experimental models, principally rats and Guinea pigs due to ease of handling and similarities with the human ear^9^. It is important to stress that the outcome of this study is reserved only to the studied sample (albino Guinea pigs), as according to Salt^21^ the interpretation of experimental studies is often complicated by the differences in cochlear dimensions found between human beings and most experimental animals.

A vast array of experimental studies has attempted to find less toxic therapies and effective means to protect the cochlea against chemical and physical insults^11^. Similarly to our study, safety-related aspects regarding the use of antibiotic therapy have also been considered.

## CONCLUSION

No changes were observed in outer hair cell function of albino Guinea pigs treated with gentamicin 4 mg/ Kg/day and 2.5 mg/Kg/day every 12 hours for 10 and 14 days.
